# The Accumulation and Molecular Effects of Trimethylamine N-Oxide on Metabolic Tissues: It’s Not All Bad

**DOI:** 10.3390/nu13082873

**Published:** 2021-08-21

**Authors:** Emily S. Krueger, Trevor S. Lloyd, Jeffery S. Tessem

**Affiliations:** 1 Department of Nutrition, Dietetics and Food Science, Brigham Young University, Provo, UT 84602, USA; emilys.krueger@gmail.com (E.S.K.); tslloyd@gmail.com (T.S.L.); 2 Medical Education Program, David Geffen School of Medicine at UCLA, Los Angeles, CA 90095, USA

**Keywords:** western diet, trimethylamine n-oxide (TMAO), gut microbiome, metabolic tissue function, oxidative stress, metabolic diseases, obesity, diabetes, insulin resistance, insulin production

## Abstract

Since elevated serum levels of trimethylamine N-oxide (TMAO) were first associated with increased risk of cardiovascular disease (CVD), TMAO research among chronic diseases has grown exponentially. We now know that serum TMAO accumulation begins with dietary choline metabolism across the microbiome-liver-kidney axis, which is typically dysregulated during pathogenesis. While CVD research links TMAO to atherosclerotic mechanisms in vascular tissue, its molecular effects on metabolic tissues are unclear. Here we report the current standing of TMAO research in metabolic disease contexts across relevant tissues including the liver, kidney, brain, adipose, and muscle. Since poor blood glucose management is a hallmark of metabolic diseases, we also explore the variable TMAO effects on insulin resistance and insulin production. Among metabolic tissues, hepatic TMAO research is the most common, whereas its effects on other tissues including the insulin producing pancreatic β-cells are largely unexplored. Studies on diseases including obesity, diabetes, liver diseases, chronic kidney disease, and cognitive diseases reveal that TMAO effects are unique under pathologic conditions compared to healthy controls. We conclude that molecular TMAO effects are highly context-dependent and call for further research to clarify the deleterious and beneficial molecular effects observed in metabolic disease research.

## 1. Introduction

**Figure 1:**
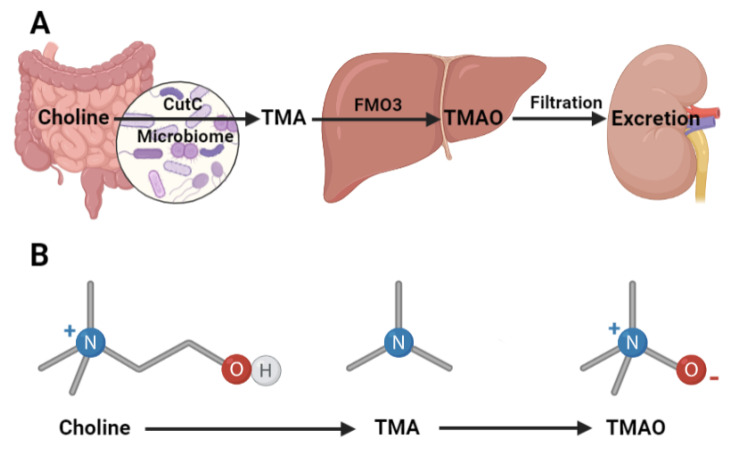
Trimethylamine N-Oxide (TMAO) Accumulation in Serum. (**A**) The microbiome-liver-kidney axis regulates TMAO production and accumulation. Choline and related compounds from dietary animal proteins and fats are metabolized by gut bacteria expressing choline utilization cluster (Cut) genes including *E. coli*. The resultant trimethylamine (TMA) is absorbed by enterocytes and metabolized by hepatic flavin-containing monooxygenase (FMO) enzymes. Serum TMAO is excreted via renal glomerular filtration and uptake by proximal tubular cells through organic cation transport proteins. (**B**) Choline, TMA, and TMAO structures. This figure was created with biorender.com.

Trimethylamine N-oxide (TMAO) chemistry has been investigated since the 1890s and TMAO was first reported in human urine samples in 1934 [1,2,3,4,5]. Urine TMAO levels have since been associated with dietary choline consumption and chronic diseases [6,7,8]. Positive and negative molecular mechanisms of TMAO have been identified in various tissues across many species [9,10,11,12,13,14,15,16,17]. The 2011 landmark metabolomics study first linked elevated serum TMAO levels to cardiovascular disease (CVD) and TMAO research in the context of chronic diseases has since grown exponentially [8]. We now know that serum TMAO is derived from choline via a gut microbiome metabolite and represents a critical factor for exploring the diet-gut-host effects on health (Figure 1) [8,18,19,20,21,22,23,24,25,26,27,28,29]. Clinical TMAO effects are most closely tied to atherosclerotic phenotypes, although there is some debate [30,31]. Resent TMAO research has recently expanded to include other chronic metabolic diseases [20,32,33,34,35,36,37,38,39,40,41,42,43,44,45,46,47]. Over-nutrition related metabolic phenotypes including insulin resistance, type 2 diabetes (T2D), obesity, metabolic syndrome, and chronic kidney disease (CKD) which are already associated with CVD have been linked to TMAO [39,44,48,49,50,51,52,53,54,55,56,57,58,59,60,61,62,63,64,65,66,67,68]. Emerging molecular level studies are beginning to elucidate TMAO’s effects on various relevant metabolic tissues; however, direct TMAO mechanisms are still unclear. While it is debated whether TMAO plays predominantly positive or negative roles in the metabolic disease contexts, TMAO is generally considered deleterious and strategies to reduce its accumulation are proposed for better CVD treatment [50,69,70,71,72,73,74,75,76]. Here we define how serum TMAO accumulates with a close look at the interaction between the diet, the microbiota, the host liver, and kidney tissues. We then explore the known TMAO effects in metabolic tissues including the liver, kidney, brain, adipose, and muscle. Finally, to further link to metabolic diseases which commonly involve poor blood glucose management, we will review how TMAO affects insulin resistance and insulin secretion. Just as the role of TMAO may differ between CVD and metabolic disease conditions, we hypothesize that it may function differently between healthy and diseased states.

## 2. TMAO Accumulation in Serum

### 2.1. Intestinal TMA Production

The source of circulating TMAO is the precursor trimethylamine (TMA) produced by the intestinal microbiome metabolism of dietary choline. High levels of quaternary amine-containing semi-essential nutrients such as choline, phosphatidylcholine, carnitine, betaine, and ergothioneine are common in a Western diet containing animal proteins and fats [69,77,78,79,80]. These foods and nutrients may or may not be directly linked to CVD independent of TMAO production [8,69,81]. Diets high in plant products, including the Mediterranean, vegetarian, and vegan diets are associated with lower circulating TMAO [50,82,83,84]. However, even healthy diets containing fish, vegetables, and whole-grain products, which measured high levels of long-chain unsaturated fatty acids can increase serum TMAO levels in patients with at cardiometabolic risk [85]. In patients with obesity, a vegan diet intervention reduced circulating TMAO levels and improved glucose tolerance presumably by reducing intake of the precursor nutrients [50]. Therefore, the first step toward serum TMAO accumulation is the consumption of prerequisite nutrients.

In a Western diet, the abundance of choline-related nutrients can surpass the absorptive capacity of the small intestine and the excess is metabolized by the large intestinal microbiota prior to absorption [86,87,88,89]. In carnitine challenged omnivorous and vegetarian subjects, TMAO production increased 10-fold in omnivores [90]. Presumably, the excess carnitine substrate was metabolized by TMA producing bacteria which were disproportionately abundant in the microbiomes of omnivores [71,91]. However, it must be noted that carnitine is not a primary pre-curser of TMA, as discussed below. Aged animal models show higher TMA absorption into the portal vein and eventual TMAO accumulation compared to the young, indicating that changes at the host enterocytes also influences serum TMAO accumulation [92,93,94].

TMA producing anaerobic bacteria have been identified by screening gastrointestinal isolates and using bioinformatic analysis [95,96,97]. These studies found that TMA is produced by facultative or obligate anaerobes across 4 phyla including Proteobacteria, Firmicutes, Actinobacteria, and Fusobacteria [98,99]. All species of the genera Desulfosporosinus and Proteus, and most species in the Enterococus, Escherichia, Klebsiella, Conlinsella, Closteridium, and Anaerococcus genera produce TMA [98,99]. These bacteria, including the well-known E. coli, C. bacterium, and C. hathewayi species express the choline utilization cluster (Cut) family of genes [98]. The glycyl radical enzyme homologue choline TMA-lyase (CutC) is activated by CutD and cleaves the C-N bond in choline to produce TMA and acetaldehyde [99,100,101]. The oxygenase Rieske 2S-2Fe cluster-containing enzymes CntA and CntB have a broader affinity for secondary dietary substrate including carnitine and betaine [97,102]. Although some TMAO research is performed in carnitine supplemented models, these generally report findings contrary to choline feeding studies presumably because carnitine is a poor TMA precursor because it is generally metabolized to γ-butyrobetaine [91,97,103,104]. It must be noted that the gene pair YeaW and YeaX may further modify γ-butyrobetaine to TMA because of its substrate promiscuity [97,105]. Therefore, while TMA can be generated from various dietary quaternary amine-containing semi-essential nutrients, most studies focus on choline metabolism via CutC and CutD. Because TMA-producing species and genes are now well-defined, some studies propose personalized strategies based on the TMA productivity of an individual’s microbiome [96]. These strategies include drug and dietary interventions generally aimed at reducing TMAO levels due to their association with CVD and other chronic diseases [78,106,107,108,109,110]. While the common insulin sensitizing drug Metformin decreases gut bacterial TMA production and eventual serum TMAO levels in T2D model mice [111], iodomethylcholine and 3,3-dimethyl-1-butanol directly inhibit the microbiota TMA [78,112]. Although the substrates for the TMA-producing enzymes are largely dietary, it must be noted that the choline moiety is also present in endogenous bile acids which are absorbed and recycled [113,114]. Therefore, the presence of TMA-producing species in the microbiota may be more critical to overall TMAO production and accumulation than the dietary make-up [78]. This conclusion highlights how dietary interventions to reduce meat, milk, and eggs is not always effective at reducing serum TMAO levels. Together these studies establish that microbiota metabolism of choline related nutrients produces the obligatory TMA precursor for TMAO production.

Convincing antibiotic studies further validate the primary role of the intestinal microbiome in TMAO production and accumulation. When the microbiome is intact and diets are supplemented with substrates, circulating TMAO levels increase predictably [36,91]. When the microbiome is absent, as in gnotobiotic or antibiotic treated animals, TMAO levels and its subsequent effects are blocked [42,91,115,116,117]. In humans, broad-spectrum antibiotics significantly reduce serum TMAO levels which recover after treatment is withdrawn [118]. When gnotobiotic animals are colonized by TMA producing human gastrointestinal isolates, serum TMAO levels increase [95]. These levels also increase when TMAO is supplemented directly [36]. Fish meat containing high levels of TMAO increased serum levels within 15 min of consumption, indicating that TMAO can also be absorbed at the small intestine [19]. In vitro research shows that TMA can be oxidized by reactive oxygen species (ROS) which may play a role during intestinal inflammation [119,120]. Hence, many animal studies use TMAO supplementation to effectively model elevated levels reported in the clinical setting; however, this model is most appropriate for research investigating high fish consumption or inflammatory bowel disease. Other radiolabeled TMAO feeding studies show that TMAO retroconversion to TMA by the microbiome is also possible [21]. This retroconversion is the hallmark of trimethylaminuria, also called fish odor syndrome because this reaction is also present in rotting fish [121,122,123,124,125]. Together, these findings demonstrate that while dietary TMAO can be directly absorbed at the small intestine, microbiome metabolism of choline at the large intestine generates the bulk of TMA levels which are absorbed and delivered to the liver via the portal vein.

### 2.2. Hepatic TMAO Production

The flavin-containing monooxygenase (FMO) enzyme family, which is highly expressed in the liver, converts TMA to TMAO. TMA is taken up by hepatocytes and oxidized to TMAO by FMO enzymes which function in the same way as cytochrome P450 oxidoreductases [126]. FMO enzymes are located at the endoplasmic reticulum (ER) and oxidize a broad range of neutrophilic substrates with flavin adenine dinucleotide (FAD) and nicotinamide adenine dinucleotide phosphate (NADPH) cofactors [126]. Their capacity to oxidize TMA was established in the 1960s through fish cell biology research and has since been identified as a liver-specific process in many vertebrates [4,5]. Humans have FMO enzymes 1 through 5 with each isoform expressed in a tissue dependent manner. FMO3 and 5 are liver-specific and have the highest overall expression [32,126,127,128,129]. FMO3 has a 10-fold higher TMA specific activity than FMO1 in vitro [32,130]. Baseline FMO3 expression is contingent on age and sex such that adults have higher expression than children younger than 6 years old and females have higher levels than males [32,48,131]. Therefore, a common research strategy is to use adult female subjects to investigate FMO3 overexpression and inhibition predictably increases and reduces serum TMAO levels respectively [32]. Serum TMAO levels peak 4 h after nutrient precursor consumption illustrating the stepwise TMAO formation via microbial metabolism, intestinal absorption, and hepatic oxidation [132]. TMAO can be excreted from hepatocytes into the serum by organic cation transporters or it may remain intracellular to influence hepatocyte metabolic functions as will be discussed below [133]. Therefore, after microbiota metabolism of dietary nutrients, FMO3 represents the final step of host mediated TMAO production.

The diet influences TMAO formation not only by providing substrates to the microbiome for TMA production, but also by indirectly regulating FMO3 expression. This control is evident in choline, carnitine, and TMAO supplementation studies, which predictably increased circulating TMAO levels, but also enhanced FMO3 expression [36,91]. This elevated expression may be compensatory due to increased TMA substrate load but may also represent FMO3 expression regulation by TMAO [36]. Mice models of over-nutrition have elevated FMO3 expression [36,93,117,134]. Conversely, one hepatocyte cell culture study showed that free fatty acid treatment inhibited FMO1, 3, and 5 expression [135]. Still, either by providing the TMA precursor or by some other indirect regulating mechanism, the diet alters FMO3 expression.

The diet further affects FMO3 expression through metabolic hormone regulation. Glucagon and corticosteroids, which drive anabolic pathways in the fasted state, induce FMO3 expression up to 14-fold and significantly increase TMAO accumulation [48]. Insulin induces anabolic pathways during the fed state and suppresses FMO3 expression by 60% in primary rat hepatocytes [48]. Inhibition of phosphoinositide 3-kinase (PI3K), a downstream insulin signaling effector, blunted insulin’s inhibitory effect [48]. Non-biased metabolomic studies identified TMAO and FMO3 as targets of insulin signaling [48]. In fact, FMO3 expression in mice modelling insulin resistance surpassed expression rates under glucagon stimulation and illustrate that insulin’s inhibition of FMO3 eclipses the induction observed under high glucagon conditions [48,136]. Conversely, insulin treatment did not affect FMO1 activity in healthy wildtype rats [137]. This discrepancy in insulin’s regulation of TMAO production supports the hypothesis that TMAO may work differently under healthy conditions compared to insulin-resistant conditions. In conclusion, the most convincing studies show that insulin signaling is sufficient to impede FMO3 expression and TMAO production which are induced by glucagon during the fasted state.

Clinical data from insulin resistant patients confirm that metabolic hormones regulate FMO3 expression. Obese patients fasting prior to bariatric surgery had higher FMO3 levels compared to control patients [48]. Of the patients, 79% were diabetic and most were treated with insulin sensitizing drugs. These results strongly confirm that insulin resistance combined with glucagon signaling during fasting drives FMO3 expression. However, unlike the animal studies [48,136], improved insulin signaling due to drug interventions could not overcome these effects. This study speaks to the dysregulated hormonal condition of T2D where insulin resistance may limit an important check on FMO3 expression and allow elevated TMAO production and accumulation [48].

A final known regulator of FMO3 expression is the bile acid-activated farnesoid X receptor (FXR). FXR loss and gain of function experiments link FMO3 transcriptional regulation to lipogenic pathways [32,48]. BA-bound FXR increases FMO3 expression 17-fold and overexpression of FXR induces FMO3 expression in a dose-dependent manner [48]. Studies identified an FXR response element in the FMO3 promoter and FXR regulation was eliminated when this region was mutated [32,48]. These results confirm that FXR directly binds the FMO3 promoter in animals consuming diets ranging from over-nutrition to healthy controls in [32,48]. Together these studies show that hepatic TMAO production by FMO3 is controlled by BA-bound FXR transcriptional regulation along with insulin hormonal regulation.

### 2.3. Renal TMAO Filtration

Kidney function plays a final role in managing circulating TMAO levels. In healthy subjects, TMAO is efficiently excreted in the urine to maintain serum levels below about 10 μM [60,71,129,138]. Glomerular filtration and uptake by proximal tubular cells through organic cation transport proteins regulates circulating TMAO and prevents excess accumulation [133,139,140]. While small amounts may be secreted as TMA after retroconversion, renal FMO3 and 1 expression accounts for over 96% being excreted as TMAO [128,129,141,142]. Although urine TMAO levels correlate to serum levels and are often reported in TMAO research, the best studies report serum levels which better represents the combined TMAO regulation from the microbiome, liver, and kidney [90]. Indeed, in a case of elevated protein intake resulting in elevated TMAO production, renal excretion of TMAO was unexpectedly decreased due to individualistic factors including these regulatory tissues [143]. Therefore, proper kidney function represents the final check on serum TMAO accumulation.

**Figure 2:**
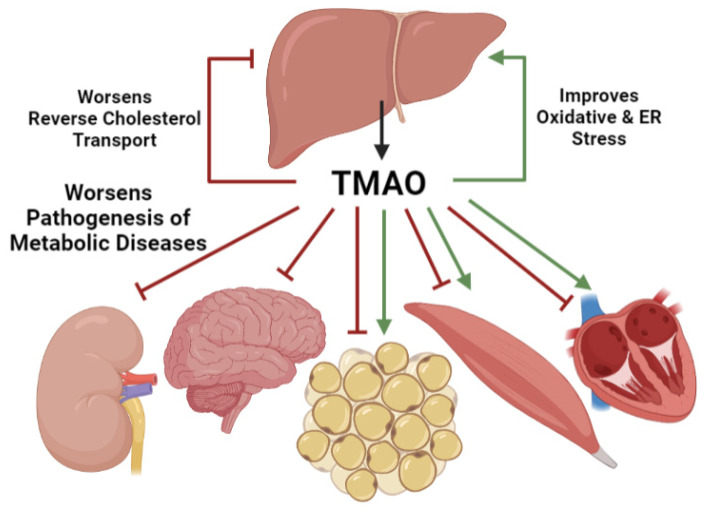
TMAO Effects on Metabolic Tissues. Elevated serum TMAO is generally associated with metabolic diseases and molecular effects are mainly observed in pathogenic and not healthy conditions. Molecular TMAO effects are best defined in hepatocytes where it worsens reverse cholesterol transport and drives non-alcoholic liver disease and gallstone formation. However, TMAO also improves oxidative and endoplasmic reticulum (ER) stress associated with hepatic insulin resistance. While poor renal function drives TMAO accumulation, it in turn aggravates chronic kidney disease. Although direct mechanisms are unclear, TMAO is associated with cognitive diseases including autism spectrum disorder and Alzheimer’s disease. Interestingly, TMAO is liked with increased adiposity associated with obesity and type 2 diabetes (T2D), but it also reduces adipocyte ER stress. In cardiovascular disease research, TMAO may increase or decrease cardiac muscle hypertrophy associated with heart failure. In skeletal muscle, TMAO benefits enzyme kinetics but is debated to drive insulin resistance. Therefore, TMAO effects may be positive or negative depending on the context of metabolic disease and molecular mechanisms are not well understood. This figure was created with biorender.com.

When kidney function is impaired, as in CKD associated with T2D, elevated TMAO levels range from 20 μM to greater than 100 μM [49,106,144,145,146,147,148]. CKD progression is associated with elevated TMAO levels such that stage 4 and 5 CKD patients had disproportionately elevated serum TMAO levels compared to stage 1 to 3 CKD patients [145,148,149]. Deleterious kidney mechanisms including reduced glomerular filtration rate, fibrosis, and loss of tubular function are associated with elevated serum TMAO [55,150,151]. One study on coronary artery disease patients showed a relationship between TMA levels and glomerular filtration rate [152]. In treating CKD, loop diuretics aggravate the elevated TMAO levels whereas renal transplantation dramatically reduces levels, highlighting that proper renal filtration is sufficient to limit TMAO accumulation [67,153,154]. Together, these data demonstrate that after precursor TMA production by the intestinal microbiome and hepatic conversion to TMAO by FMO3, renal filtration is the final regulator of circulating TMAO levels. Differences in any step of this TMAO production and accumulation may explain the extreme variability in serum TMAO levels observed clinically [155]. Despite this variation, accumulated serum TMAO can affect metabolic functions in tissues throughout the body (Figure 2).

## 3. TMAO Effects on Metabolic Tissues

### 3.1. TMAO Effects on Liver Function

Because TMAO is produced by hepatic FMO3, potentially higher local concentrations affect liver metabolic functions. Because of the association between elevated serum TMAO levels and various liver diseases, TMAO effects are generally considered deleterious, however TMAO is debated to have beneficial roles in some cellular contexts. Non-alcoholic fatty liver disease (NAFLD) is characterized by hepatic lipid accumulation and is associated with obesity, T2D, and insulin resistance which are also linked to elevated TMAO levels [48,156,157]. NAFLD patients and high fat high cholesterol diet fed animal models demonstrate elevated TMAO compared to healthy controls [134,158]. Hepatic TMAO levels are associated with markers of poor liver function including steatosis, serum bile acid levels, and inflammation in patients stratified by disease severity [159]. Gallstones and primary sclerosing cholangitis involve dysregulated hepatic bile formation and are associated with increased serum TMAO levels [36,160]. Cholangitis patients with serum TMAO levels over 4 μM have shorter liver transplant free survival rates [160]. Although these studies do not demonstrate a mechanistic role for TMAO at the liver, they provide the clinical rational for molecular research on TMAO effects on liver function.

Mouse liver disease models more directly link TMAO to altered hepatic function. In these models, over-nutrition induces disease phenotypes including increased hepatic lipogenesis rates, steatosis, serum aspartate transaminase or alanine transaminase levels, and insulin resistance, which are associated with elevated serum and urine TMAO levels [134,156]. While most studies use female mice, some report TMAO accumulation and aggravated liver disease in male mice which are known to have a lower basal expression of hepatic FMO3 and lower average TMAO production than females [32,48,142]. Despite the potential inhibition from androgen hormones in these male mice, the high fat diet significantly increased TMAO levels and escalated liver disease phenotypes [134]. Interestingly, TMAO supplementation in chow fed male mice did not exhibit altered steatosis scores [134]. Similarly, in healthy elderly female patients taking carnitine supplements, plasma lipid and inflammation markers were not associated with TMAO accumulation, presumably because carnitine is a poor TMA pre-curser as described above [103,104]. These studies on healthy subjects may also demonstrate that TMAO may not influence hepatic functions in a disease-free environment [103,104,134]. In gallstone susceptible mice, a high cholesterol lithogenic diet combined with TMAO supplementation demonstrated increased gallstone formation [36]. Even gallstone resistant mice developed gallstones on the same dietary regimen yielding 7 μM serum TMAO levels [36]. These various liver disease model studies further associate elevated TMAO with poor liver function and motivate a deeper look at TMAO molecular effects.

The liver is a major insulin sensitive tissue assisting with blood glucose management. In healthy conditions, proper insulin signaling upregulates glycogen and lipid synthesis to reduce blood glucose levels. TMAO is however linked to insulin resistant conditions resulting in elevated blood glucose levels. In mice modeling hepatic insulin resistance, TMAO reduction by FMO3 knockdown prevented hyperlipidaemia [48]. TMAO supplemented high fat diet fed mice had decreased glycogen synthesis and FMO3 knockout mice with reduced TMAO levels had increased synthesis which connects TMAO to hepatic insulin resistance [161,162]. Gluconeogenic genes glucose 6-phosphatase (G6pase) and phosphoenolpyruvate carboxykinase (PEPCK) typically suppressed by insulin were concomitantly upregulated in the liver, muscle, and adipose tissue of TMAO supplemented mice [161]. Furthermore, FMO3 overexpression in hepatic cell cultures increased glucose secretion, which is also typically suppressed by insulin [162]. Finally, TMAO induction of insulin resistance was more directly measured by reduced expression of the insulin signaling cascade including insulin receptor substrate 2 (IRS2), PI3K, RAC-β serine/threonine-protein kinase (AKT) and glucose transporter 2 (GLUT2) [161]. These studies demonstrate that TMAO drives T2D associated insulin resistance by downregulating hepatic insulin signaling.

#### 3.1.1. TMAO and Reverse Cholesterol Transport

The liver is essential for lipid metabolism and regulates cholesterol levels. After dietary lipids, including cholesterol, are trafficked as apolipoproteins to deliver substrates to target tissues, the excess returns to the liver through reverse cholesterol transport (RCT). This cholesterol is metabolized to bile acid stored at the gallbladder until secreted for intestinal dietary lipid emulsification, digestion, and absorption. Bile acids are either reabsorbed by enterocytes or excreted in the feces, an important cholesterol efflux route. Impaired RCT is associated with CVD and NAFLD and increased bile cholesterol content is connected to gallstone formation and elevated TMAO levels are linked with both pathogenic pathways [36,51,91,117]. Conversely, intestinal TMAO effects relating to RCT are beneficial and are discussed further below. Rodent FMO3 overexpression elevated plasma cholesterol levels by 20% and FMO3 knockdown reduced plasma and hepatic lipid levels indicative of NAFLD [117,162]. Since TMAO reduction by FMO3 knockout decreased bile cholesterol content, elevated TMAO presumably drives gallstone formation [32]. Most liver-specific findings indicate that altering TMAO levels is sufficient to alter cholesterol flux through RCT via bile acid and cholesterol synthesis. Therefore, these studies set the precedence for using NAFLD and gallstone formation models to investigate the molecular pathways involved in hepatic TMAO effects.

The RCT rate and hepatic cholesterol pool is controlled by cholesterol transporters and synthesis enzymes, which are altered by TMAO. Low-density lipoproteins and high-density lipoproteins are a major source of the hepatocyte cholesterol pool, and their receptors include low-density lipoprotein receptor (LDLR) and scavenger receptor class B type 1 (SRB1). Alternatively, cholesterol and bile acids can be taken up through transporters sodium/taurocholate co-transporting polypeptide (NTCP) and organic-anion-transporting polypeptides (Oatp1 and Oatp4), which are upregulated in hepatocytes cultured with supra-physiological 250 μM TMAO [36]. This upregulation by TMAO is validated in gallstone-susceptible mice with 6 μM serum TMAO and in gallstone patients with 3.3–4 μM serum TMAO [36]. One study on NAFLD patient biopsies also demonstrated no change in NTCP levels [134]. Despite some inconsistency, the evidence supporting that TMAO increases hepatic cholesterol uptake is convincing because results are validated in human subjects with both extremely elevated and slightly elevated TMAO levels [36].

Hydroxymethylglutaryl-CoA synthase 1 (HMGCS1) is the key regulated enzyme for endogenous cholesterol synthesis which contributes to deleterious hepatic cholesterol accumulation. Mice with reduced TMAO levels from FMO3 or FMO5 deficiency had downregulated HMGCS1, supporting the hypothesis that TMAO increases cholesterol synthesis [163]. Similarly, triacylglyceride synthesis was increased with FMO3 overexpression and decreased with knockout [162,163]. Furthermore, malic enzyme 1, which produces NADPH used in lipid biosynthesis, was decreased in FMO5 knockout mice [163]. Increased hepatic cholesterol can be further metabolized to BA. Bile acid synthesis is primarily regulated by cholesterol α-hydroxylases (Cyp7a1 and Cyp27a1). Male wildtype high fat diet fed mice and NAFLD patients present elevated serum TMAO and increased Cyp7a1 expression [134]. Cyp7a1 levels are also elevated in female apolipoprotein E knockout mice on a chow diet with choline supplementation [164]. These studies expand the lipogenic role for TMAO to include the production of endogenous lipids cholesterol, triacylglycerides, and bile acids. Therefore, TMAO increases the hepatic cholesterol pool by upregulating lipid uptake and synthesis which validates the aggravated hepatic lipid accumulation observed clinically.

Cell culture, animal, and clinical research shows that TMAO alters canalicular cholesterol and bile acid transport to promote gallstone formation. The ATP binding cassette transporters (Mrp2, BSEP, ABCG5, and ABCG8) are located at the cholesterol-rich apical cell membrane on hepatocytes. Their sterol and bile acid substrates are stored in the gallbladder until they are secreted to aid in intestinal lipid digestion and may eventually be excreted in the stool. ABCG5 and ABCG8 primarily transport sterols, including cholesterol, whereas BSEP transports bile salts. Expression of these transporters control the cholesterol to bile acid ratio at the gallbladder which when increased can gallstone formation. Hepatocytes cultured with TMAO had increased ABCG5 and ABCG8 levels, while TMAO reduction by FMO3 knockout decreased expression [36]. Data from genetic or over-nutrition induced gallstone formation models support this finding [36,117]. Similarly, patients with gallstones had increased ABCG5 and ABCG8 levels and decreased BSEP levels [36]. FMO3 loss or gain-of-function experiments in cholesterol fed mice demonstrated that TMAO induces ABCG5 and ABCG8 expression, and presumably increases bile cholesterol content to drive gallstone formation [117]. Together, these data illustrate that TMAO induces canalicular cholesterol transport in preference over BAs and promotes gallstone formation.

In contrast to the deleterious TMAO effects on hepatocytes, TMAO may trigger interesting beneficial changes at the intestines related to RCT and cholesterol efflux [51]. Dietary cholesterol and endogenous bile acids in the lumen of the intestine are absorbed by enterocytes, packaged into apolipoproteins, and delivered to the lymphatic system. The transporter niemann-pick C1-like 1 (Npc1l1) is critical for cholesterol absorption into enterocytes. While there is some debate, TMAO generally reduces Npc1l1 which presumably combats high blood cholesterol levels associated with CVD and CKD [117,162]. TMAO supplementation in high cholesterol diet fed mice reduced Npc1l1 levels and cholesterol absorption rates by 26% [91,165]. Conversely, cholesterol may be transported out of enterocytes and into the lumen via ABCG5 and ABCG8 for fecal excretion. As shown in hepatocytes, TMAO supplementation increased enterocyte ABCG5 and ABCG8 expression and decreased TMAO from FMO3 knockout blunted expression [91,162,165]. While in hepatocytes this elevated expression increased bile cholesterol content and promoted gallstone formation, in enterocytes this elevated expression may benefits cholesterol efflux [117,166]. Therefore, intestinal TMAO effects could combat chronic diseases associated with cholesterol accumulation including obesity, CVD, NAFLD, and gallstone formation. It must be noted however that since TMAO is produced at the liver and not in the intestine as described above, results on enterocyte changes from TMAO feeding studies my not be physiologically relevant except when high fish consumption or inflamed bowel disease is considered.

A final regulator of hepatic cholesterol metabolism affected by TMAO is FXR which can be bound by various agonist or antagonist BA. Because TMAO influences bile acid formation, it alters the FXR ligand abundance which ultimately impacts its regulation of lipogenic pathways, including cholesterol metabolism and bile acid synthesis. Agonist-bound FXR activates small heterodimer partner (SHP) which inhibits the lipogenesis regulator sterole regulatory element-binding protein 1 (SREBP1c) to reduce cholesterol and bile acid synthesis [167,168]. Conversely, antagonist-bound FXR inhibits SHP, leaving cholesterol and bile acid synthesis active. Most studies show that TMAO drives the agonist-bound FXR inhibition of SREBP1c and the bile acid synthesis enzyme Cyp7a1 [91,117,164]. However, one study using cellular, animal, and clinical data showed that TMAO increased lipogenesis similar to antagonist-bound FXR studies [134]. Because of this confusion, more research is needed to decipher the positive or negative effects that TMAO exerts on FXR regulation via altering its ligand abundance.

In the context of liver cholesterol metabolism during pathogenic conditions, studies generally show that TMAO is deleterious to liver function. In terms of hepatic cholesterol uptake, synthesis, and canalicular transport, TMAO plays a negative role. It aggravates NAFLD by increasing hepatic cholesterol content and exacerbates gallstone formation by elevating biliary cholesterol content. In experiments surrounding bile acid synthesis and bile acid-bound FXR transcriptional activity there are conflicting results. Interestingly, cholesterol absorption and excretion transporters at the enterocyte are regulated by TMAO in a similar manor to hepatocytes, but the phenotype may be considered beneficial. While more research on the RCT system is needed to clarify some details, hepatic molecular studies typically highlight TMAO as an aggravator of poor liver cholesterol metabolism leading to NAFLD and gallstones.

#### 3.1.2. TMAO, Oxidative Stress, and Endoplasmic Reticulum Stress

Contrary to the previous reports that TMAO aggravates liver diseases, some studies show that TMAO may beneficially combat cellular stresses. There is a close relationship between ER stress and oxidative stress exerted by deleterious ROS. Both stresses are clearly implicated in chronic liver diseases [116,169,170,171]. Although it is well established that TMAO increases oxidative and ER stress levels in arterial tissue, studies in other tissue types including hepatocytes report a surprising beneficial role for TMAO [94,116,172,173,174]. In fish, TMAO is an important osmoregulator for maintaining proper cell volume during osmotic pressure [175,176]. The promoter region of FMO genes in many fish species have putative osmoregulatory response elements [177]. When accumulated, urea and other ER stressors cause protein denaturation or mis-folding, against which TMAO is protective [175,178,179,180,181,182]. Thus, TMAO is also defined as a protein folding chaperone which reduces ER stress and the resultant unfolded protein response (UPR) leading to apoptosis [16,183]. Recent molecular dynamics studies show that TMAO acts as a surfactant between the folding protein and its aqueous environment by reorganizing the hydrogen bond network to selectively stabilize proteins experiencing structural collapse [17,182,184,185]. Indeed, many molecular experiments investigating ER stress and the UPR, including those using primary human tissues, use TMAO treatments as experimental controls [186,187,188]. As a chaperone, TMAO is, therefore, a strong candidate for combating oxidative ER stress and the UPR relevant to many chronic diseases including insulin resistance [189].

Cell culture and animal studies validate this beneficial TMAO effect in hepatocytes modeling various metabolic diseases. Over-nutrition-induced NAFLD model mice supplemented with TMAO had reduced liver damage as measured by serum aspartate transaminase and alanine transaminase levels [165]. TMAO also improved cholesterol metabolism by reducing liver and serum cholesterol and serum low density lipoprotien levels which correlated with downregulated ER stress, UPR, and apoptosis genes [165]. Similarly, palmitate induced chronic ER stress associated with hepatic insulin resistance was reversed by short and long-term TMAO treatments [171]. However, one palmitate induced oxidative stress study showed that TMAO reduction by FMO3 knockdown reduced palmitate induced ROS levels by 20% [135]. In high cholesterol diet fed mice, TMAO supplementation reduced ER stress and inflammation [117]. As discussed earlier, this study investigated the RCT in FMO3 knockdown mice and identified a reduction in oxysterol availability. Since oxysterols are a bile acid ligand for the transcription factor liver X receptor (LXR), they measured the bile acid-bound LXR transcriptional suppression of the inflammation and ER stress response in hepatocytes. In the absence of TMAO, inflammation was increased by macrophage-derived proinflammatory cytokines and ER stress genes associated with fatty acid-induced stress. FMO3 overexpression downregulated the relevant deleterious genes [117]. Despite some debate, in vitro and in vivo studies generally demonstrate that TMAO protects against hepatic ER stress and the associated inflammation and apoptosis at the transcriptional level [117,165,171].

One robust study identified that TMAO directly binds an ER stress response protein which may underpin the beneficial TMAO effects described. Using healthy and ER stress animal and hepatocyte models, this study showed that TMAO upregulated UPR and apoptosis genes which were reversed by FMO3 knockdown [136]. Furthermore, radiolabeled TMAO co-precipitated with the protein kinase R-like ER kinase (PERK) indicating direct binding between TMAO and the UPR regulating transcription factor. Seemingly, these findings contrast with the earlier results showing that TMAO reduced ER stress, UPR, and apoptosis gene expression [165]. However, activation of the UPR during acute ER stress by PERK is debatably adaptive [190]. Acute PERK activation reduces ROS and preserves proteostasis, redox homeostasis, and mitochondrial function [191,192,193,194]. Indeed, this study is the first to identify a direct binding effect of TMAO during short-term ER stress conditions [136]. Together with other studies, these findings demonstrate a uniquely beneficial role for TMAO in healthy and pathologic hepatocytes [117,165,171].

TMAO effects on ER and oxidative stress are of particular interest to metabolic disease research. Reducing ER stress is sufficient to normalize phenotypes including hyperglycemia and insulin sensitivity by improving insulin action at the liver [195]. These hepatocyte studies elucidate that TMAO reduces ER and oxidative stress and limits the UPR and apoptosis. Other chronic diseases implicated by these cellular stresses include CVD, NAFLD, obesity, T2D, CKD, and cognitive diseases where TMAO effects the various tissues relevant to each disease as discussed below. The molecular studies mentioned here provide evidence through short and long-term studies with low and high TMAO concentrations using in vivo, ex vivo, and in vitro experiments. Therefore, strong evidence supports the hypothesis that, similar to its beneficial effects in fish cell biology, TMAO plays a beneficial oxidative and ER stress-mitigating role in human physiology. Further research will help to elucidate the various health conditions where this beneficial TMAO effect is most relevant to clinical outcomes.

### 3.2. TMAO Effects on Kidney Function

While TMAO can accumulate due to poor filtration during CKD, some studies suggest that TMAO aggravates CKD in turn. CKD animal models are used to investigate potential mechanisms that underpin TMAO elevation beyond that expected with impaired kidney during CKD [55,147,196]. The TMAO mechanisms related to CKD involve crosstalk between the TMAO producing tissues including the intestinal microbiome, liver, and kidney. Fecal transplants from CKD or healthy patients to antibiotic treated mice altered the microbiome with a bias toward opportunistic TMA producing species [147]. CKD mouse models have upregulated hepatic FMO3 which highlights the liver involvement in CKD [146]. Hepatic FMO3 genetic allelic variants corresponding with elevated TMAO production are also associated with CKD [197]. FMO3 also has higher substrate affinity in CKD animals, suggesting that elevated serum TMAO levels may be dependent on hepatic function independent of the low filtration rates expected during CKD [145,197]. FMO3 kinetic studies utilizing FMO3 inhibitors and activators with CKD or healthy control serum found that CKD serum contains an unknown compound which induces FMO3 [145]. While TMAO is a candidate for this regulatory effect, its involvement was not directly measured [145]. Together these studies allude to potential inter-tissue signals driving TMAO accumulation beyond the levels expected during CKD. But further studies are required to establish kidney-specific molecular actions of TMAO. 

Molecular studies are required to elucidate mechanisms linking TMAO and CKD development. Clinical studies categorize TMAO as a uremic toxin which induce pathophysiologic changes implicated in CKD [144,154,198]. Increased TMAO levels at a healthy baseline predicts future CKD development and 5-year mortality risk after controlling for traditional CKD risk factors [199,200]. In over-nutrition animal studies, choline or TMAO supplementation raised serum TMAO levels which corresponded with CKD phenotypes including collagen deposition, tubulointerstitial fibrosis, and kidney injury markers [150,173]. The TMA production inhibitors iodomethylcholine and 3,3-dimetyl-1-butanol reduced TMAO levels in CKD and obesity mice models and improved renal function by suppressing tubulointerstitial fibrosis and collagen deposition [106,173]. While other clinical studies argue no relationship between TMAO and CKD [201], or only report a deleterious association [55], these molecular studies begin to define kidney-specific TMAO effects which aggravate CKD pathogenesis.

Contrary to the deleterious TMAO effects observed in CKD, TMAO shows positive and negative effects on kidney function in the context of CVD. During a 58-week study on rats modeling heart failure, all the rats supplemented with TMAO with about 40µM serum levels survived whereas 3 of the un-supplemented group died from ischemic stroke or lung edema [202]. This increased survival corresponded with increased diuresis. Although protein expression across the renin-angiotensin pathway was altered in the TMAO group, this did not affect sodium or potassium tubular transport. Therefore, the beneficial diuretic effect of TMAO was attributed to the osmotic activity of TMAO which decreased the reabsorption of water [202]. In atherosclerosis model mice, TMAO inhibition protected against poor renal phenotypes and suggests that inhibiting microbiota TMA production is a potential treatment for renal damage during CVD [203]. These mixed TMAO effects possibly depend on the animal model used which highlights how careful selection of animal models is critical to investigating clinically relevant molecular effects of TMAO. Together these studies illustrate that TMAO is generally deleterious for CKD, but its effects on renal damage during CVD are still unclear.

### 3.3. TMAO Effects on Brain Function

TMAO crosses the blood–brain barrier, making it a candidate for influencing cognitive function and neurological diseases [93,129,204,205,206]. Research on the gut–brain axis reveals that microbiome alterations accompany changes to the cardio-sympathetic nervous system, the central nervous system, and brain chemistry [207,208,209]. Many bacteria species and their metabolite products regulate neurotransmitter expression associated with physiological and psychological stress [209,210,211]. Clinical and animal research on aged individuals highlight a connection between oxidative stress or inflammation and TMAO during age-related microbiome remodeling and cognitive deficiency [93,94,116,205,211,212,213,214]. Conversely, TMAO treatment on hippocampal sections induced oxidative and ER stress [93]. Studies on traumatic brain injury demonstrate that TMAO reduced expression of a protective antioxidant enzyme in the hippocampus, whereas treatments for reducing the injury reduced TMAO [214,215]. One study supports fecal transplants as a means of reducing TMA and TMAO production to treat stroke patients [215] Others report no relationship between TMAO and oxidative stress in healthy human or animal subjects and, therefore, highlight that TMAO’s deleterious cognitive effects may be dependent on an already diseased condition [214,216,217,218]. Interestingly, TMAO alleviated neuronal dysfunction by abrogating ER stress in diabetic neuropathy models [219]. These studies further validate that TMAO effects on neuronal tissue are context-dependent.

Cognitive disease studies demonstrate a tandem increase in oxidative stress and TMAO concentration and suggest deleterious TMAO effects at the brain. Autism spectrum disorder is associated with microbiome composition changes, inflammation, and oxidative stress [220,221,222,223]. Autism spectrum patients with higher severity scores have higher serum TMAO levels [224]. Animal models show TMAO supplementation disrupts the blood brain barrier by decreased tight junction proteins [181,225]. The heat shock protein 70 is a degradation regulator of proteins damaged by oxidative stress in barrier cells where disruption is implicated in many cognitive diseases [226,227]. Conversely, the protein folding chaperone capacity of TMAO may prevent aggregation of proteins associated with other neurodegenerative diseases including Parkinson’s, dementia, or Alzheimer’s disease [228,229,230,231]. However, it must be noted that the in vitro studies investigating this potential use supra-physiological levels of TMAO ranging from 100 mM to 2M [228,229]. Thus, because the clinical relevance of beneficial TMAO effects on neurodegenerative disease is not well established, reducing TMAO levels seems a more promising strategy for slowing the progression of neurological diseases.

TMAO is a biomarker in Alzheimer’s disease and studies identify a molecular link between TMAO, oxidative stress, and poor neuronal health [205]. An Alzheimer’s disease computational model ranked TMAO as the top associated metabolite and proposed that it may compound with other genetic and neurological factors during pathogenesis [29]. Nine molecular pathways linked elevated TMAO and Alzheimer’s disease, including various neuronal pathways, lipid and protein metabolism, the immune system, and ephrin receptor signaling [29]. Ephrin receptors are a subfamily of receptor tyrosine kinases which are integral to the function of secretory cells, including neurons and pancreatic insulin secreting β-cells [232,233,234]. Therefore, alterations in the ephrin forward signaling could link TMAO to Alzheimer’s disease and diabetes. Downstream, the metabolic regulator protein mechanistic target of rapamycin kinase (mTOR) is inhibited in the neurons of TMAO supplemented aged mice [235]. Following mTOR inhibition, increased oxidative stress and mitochondrial impairments lead to synaptic damage in the hippocampus which culminated in reduced spatial working memory indicative of the aged cognitively deficient phenotype [235]. Another metabolic regulator protein peroxisome proliferator activated receptor α (PPARα) is implicated in AD, T2D, and NAFLD [236]. FMO3 kockout mice had decreased hepatic PPARα expression [162]. Although this study did not investigate brain tissue levels, the hepatic results expand the known TMAO effects on metabolic TFs expression. While direct metabolic TMAO mechanisms are not yet established in brain tissues, these studies demonstrate that TMAO inhibits integral neuronal processes via transcription factor inhibition, oxidative damage, and altered lipid metabolism, which culminates in age-related neurodegenerative disease progression.

### 3.4. TMAO Effects on Adipose Function

Elevated TMAO increases adiposity associated with various metabolic diseases whereas decreased TMAO triggers beneficial changes in adipose tissue [132,237,238]. In humans and mice, TMAO levels increase with body mass index and visceral adiposity [237,239]. TMAO levels over 8.2 μM predict the metabolic syndrome associated with obesity [237]. Obese mice have elevated TMAO levels [157,240] and TMAO reduction by FMO knockout produces leaner mice [157,163,241]. This decreased adiposity coincided with increased metabolic flexibility in the white adipose tissue which is more typical in brown adipose tissue [157]. Furthermore, FMO3 knockout prevented obesity in insulin resistant high fat diet fed mice and increased brown adipocyte gene expression [157]. FMO5 knockout mice had greater leanness due to a 55% increase in fatty acid oxidation compared to controls [163]. The reduced adiposity in FMO1, 2 and 4 knockout mice was attributed to a futile cycle in triglyceride catabolism and re-esterification [241]. These results show a positive correlation between TMAO levels and adiposity where FMO3 knockout enabled a beneficial energy consuming phenotype.

Data further suggest that elevated TMAO levels enhance adipocyte insulin resistance and inflammation. Insulin signals exocytosis of adipocyte glucose transporter GLUT4 to help regulate blood glucose which is obstructed by TMAO. TMAO supplements in high fat diet fed mice modeling insulin resistance and inflammation demonstrated elevated fasting insulin levels and reduced insulin signaling cascade expression along with increased inflammation markers [161,242,243,244,245]. These markers included pro-inflammatory adipokines, which regulate insulin sensitivity in other metabolic tissues and coincide with insulin resistance and chronic low-grade inflammation [244,246,247]. These results indicate that elevated TMAO generally worsens adipocyte insulin resistance and drives inflammation during obesity.

Conversely, TMAO is linked with reduced adipocyte ER stress and improved insulin sensitivity. In one study, the pre-intervention urine TMAO levels predicted obesity through increased weight gain, body mass index, and adiposity [239]. However, after 5 weeks of a high fat diet, increased TMAO correlated with reduced adiposity. These surprising results corresponded with downregulated ER stress, lipid biosynthesis, insulin signaling, and adipocyte differentiation genes [239]. Reduced TMAO levels in FMO1, 2 and 4 knockout mice had 64% reduced GLUT4 expression indicating that TMAO levels are correlated to GLUT4 expression and may benefit insulin sensitivity [241]. Since these effects were not observed in healthy mice, this study supports the hypothesis that a stressed cellular environment elicits TMAO effects that are not apparent in healthy conditions [242]. In contrast to previous studies, these studies illustrate that TMAO can beneficially combat high fat diet-induced adiposity by improving insulin sensitivity and reducing ER stress which was further validated in other tissues including liver, muscle, and pancreatic β-cells [239]. When adipocyte studies using similar high fat diet models are aggregated, the bulk support deleterious TMAO effects, but a few purport beneficial effects. Therefore, further research is needed to clarify TMAO’s role in adipocyte function.

### 3.5. TMAO Effects on Muscle Function

Proper muscle function is vital for metabolic health and TMAO may benefit muscle tissue under cellular stress. The beneficial protein chaperone role for TMAO is investigated in muscle tissues similar to the previous hepatocyte studies. TMAO was first identified as a protein chaperone in fish where it accumulates in muscle tissue and protects cellular functions from the challenges of the deep-sea environment including osmotic pressure [180,248,249]. In animal muscle tissue enzyme kinetic studies, TMAO treatment beneficially increased lactate dehydrogenase (LDH) substrate affinity [250,251]. When urea treatment inhibited rabbit LDH activity, TMAO addition recovered it to control levels [250]. TMAO also increased chicken skeletal muscle myosin ATPase activity [252]. Again, ATPase activity was inhibited by urea and recovered by TMAO [252]. These in vitro studies demonstrate that the TMAO protein chaperone capacity is independent of evolutionary history because its effects on enzyme stability are observed in various species [10,11,12,14,15]. Because hydrostatic pressure stress is similar between deep-sea conditions and cardiac muscle contraction during heart failure, these beneficial TMAO effects on LDH are clinically relevant. However, in heart failure modeling rats, TMAO supplementation did not affect the tertiary or quaternary structures of LDH [202]. While these in vitro results highlight a potential protective TMAO effect on muscle enzyme activity, future in vivo studies may provide a more convincing connection to clinically relevant cellular conditions.

TMAO effects on cardiac muscle investigated in CVD research report variable results. While most CVD research investigates TMAO effects on atherosclerosis at the vascular tissue, some studies investigate cardiac muscle tissue effects independent of vascular damage which relate to metabolic health. Poor cardiac function phenotypes including altered ventricular wall dimensions, ejection fraction, fractional shortening, and interventricular wall thickness are worsened by TMAO [24,150,253,254]. The key cardiac muscle functions contraction and relaxation are also impaired by TMAO. Left ventricle contraction and relaxation times were prolonged in high fat high carbohydrate diet fed mice [255]. These times were recovered by TMAO reduction via the intestinal TMAO production inhibitor 3,3-dimethyl-1-butanol [255]. Primary rat cardiomyocytes cultured with 20 μM and 100 μM TMAO also had prolonged re-lengthening times and decreased fractional shortening [256]. These findings connect TMAO to heart failure [257]. Conversely, human atrial appendage biopsy tissue cultured with pharmacological concentrations of 300 µM to 3 mM TMAO had increased contractile tension and rate of relaxation [258]. This enhanced contractility was attributed to increased calcium signaling in primary animal cardiac tissue cultured with more physiological 20–100 μM TMAO [256,258]. Furthermore, TMAO supplemented rats accumulated TMAO in cardiac tissue which coincided with improved mitochondrial energy metabolism [259]. Other studies suggest that TMA, but not TMAO is deleterious to cardiac function, however TMA accumulation in cardiac tissue has not been demonstrated [92,260]. Since these studies show that TMAO can depress and enhance contractile function, it is unclear if TMAO is beneficial or harmful to cardiac muscle.

In animal models, TMAO alters cardiacmyocytes in potentially beneficial or deleterious ways. TMAO increases cardiac hypertrophy which may beneficially increase cardiac output, but can aggravate heart failure when combined with fibrosis, collagen deposition, and inflammation [254,261,262]. Cardiac fibroblasts cultured with 10 μM, 50 μM, and 100 μM TMAO had increased proliferation and viability [253]. TMAO and choline supplemented animals had increased heart weight to body weight ratios and cardiomyocyte fibrosys [23,24,150,253,254,263]. TMAO further increases the risk of heart failure by altering fuel utilization in cardiac tissue [264,265,266,267,268,269]. Cardiac biopsies from high fat high carbohydrate diet fed mice with elevated TMAO levels also showed increased fibrosis and inflammatory markers [255]. TMAO treatments correspond with elevated ROS levels and inflammation in cell culture [35,254,256]. In vascular smooth muscle, TMAO treatments induced ROS accumulation at the mitochondria and increased inflammation while downregulating the endogenous antioxidant defense system [35,270]. Other studies contradict these reports that TMAO worsens cardiac health by reporting no change in TMAO treated cardiomyocytes or smooth muscle cell viability or ROS levels [92,264,271]. Finally, one study observed beneficial effects where cardiomyocytes from TMAO supplemented hypertensive rats showed reduced hypertrophy and fibrosis [272]. Therefore, while many studies support the hypothesis that TMAO drives deleterious cardiac hypertrophy through fibrosis, inflammation, and ROS accumulation, potentially beneficial TMAO effects on cardiomyocytes are also reported.

In the metabolic context, insulin-sensitive muscle tissue function helps to regulate whole-body carbohydrate and lipid energy balance. Various studies highlight that TMAO alters skeletal muscle insulin sensitivity; however they report negative and positive effects. In TMAO supplemented high fat diet fed mice, the muscle tissue insulin signaling cascade was inhibited [161]. Gluconeogenic genes typically suppressed by insulin were upregulated in the muscle and other metabolic tissues of TMAO supplemented mice, indicating a link between TMAO and insulin resistance [161]. TMAO reduction by FMO1, 2, 4, and 5 knockout produced leaner mice with increased exercise capacity and resting energy expenditure by increasing muscle fatty acid oxidation rates which presumably links elevated TMAO levels to insulin resistance [163,241]. By contrast, in over-nutrition induced insulin resistant monkeys, elevated serum TMAO levels corresponded with skeletal muscle hyperlipidaemia indicating that insulin induced lipogenesis was functional [158]. TMAO enhanced insulin triggered glycogen accumulation in primary rat cardiomyocytes [256]. Despite some debate, the majority of data on insulin sensitivity show that TMAO drives muscle carbohydrate and lipid metabolism associated with insulin resistance and heart failure. However, because TMAO muscle tissue effects include beneficial changes to enzyme activity and contractile function, a dominant role for TMAO is debated and requires further research.

## 4. TMAO Effects on Blood Glucose Management

**Figure 3:**
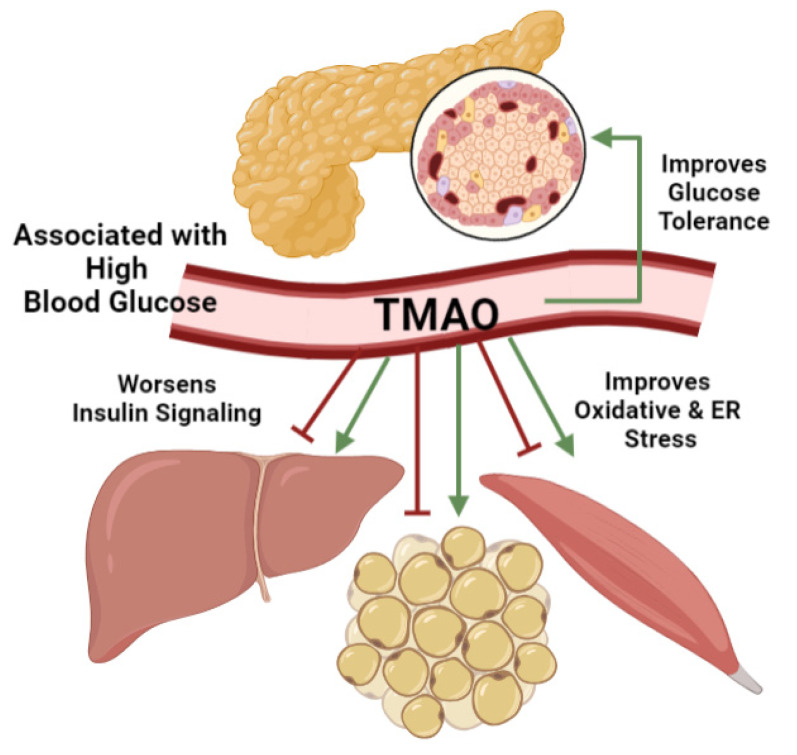
TMAO Effects on Blood Glucose Management. Elevated serum TMAO levels are associated with elevated blood glucose levels, a hallmark of metabolic diseases. Insulin resistance at target tissues or β-cell glucose intolerance can drive this phenotype. While associative studies generally link TMAO to worsened insulin resistance, the molecular evidence in hepatocytes, adipocytes, and skeletal muscles is divided. One study investigates TMAO effects on β-cell containing pancreatic islets and report improved glucose tolerance which beneficially lowers blood glucose in the T2D condition. This figure was created with biorender.com.

Poor blood glucose management is a hallmark of metabolic diseases. The hormone regulators insulin and glucagon manage metabolic functions across the body to manage blood glucose levels. Since TMAO does not alter glucagon levels, research focuses on its effects on insulin secretion and signaling (Figure 3) [162]. Situated within the pancreatic islets of Langerhans, β-cells match insulin secretion to elevated blood glucose levels to trigger glucose uptake into responsive target tissues including hepatocytes, adipocytes, and muscle tissues as discussed [273,274,275]. In T2D, elevated blood glucose stems from β-cell glucose intolerance and target tissue insulin resistance. Therefore, these mechanisms are investigated using obese patients and high fat diet fed animals which link elevated serum TMAO to metabolic diseases including obesity, gestational diabetes, and T2D [157,158,162,237,242]. In over-nutrition-induced obese mice, TMAO reduction by FMO3 knockdown reduced, and overexpression increased, body weight and fat pad adiposity [162]. This connection between TMAO and obesity is also observed clinically [50,237,238]. A vegan diet intervention in obese glucose intolerant patients reduced TMAO levels and improved postprandial blood glucose levels [50]. While these studies link TMAO to poor blood glucose management typical in metabolic diseases, a closer look at target tissue insulin resistance and β-cell glucose intolerance is necessary to identify the underlying TMAO mechanisms.

Clinical and animal studies demonstrate varying TMAO effects on insulin resistance. Although insulin inhibits FMO3 expression [48,135,136], TMAO accumulation under insulin resistant conditions may alter insulin signaling in turn. As described earlier, associative studies generally link TMAO to worsened insulin resistance phenotypes [134,156,161,163,241,242]. In obese patients, increased TMAO levels are associated with elevated fasting blood glucose and insulin levels and increased insulin resistance as assessed by homeostatic model assessment for insulin resistance (HOMA-IR) [237]. When obese patients were treated with exercise and a hypocaloric diet, TMAO levels decreased by 30% which correlated with improved insulin sensitivity and glucose disposition rates [238]. This connection between clinical insulin resistance and TMAO is matched by high fat diet fed animals [158,161]. Reduced TMAO levels in FMO3 knockdown mice reversed insulin resistance as measured by blood glucose and insulin levels [48,162]. Indeed, metabolomic studies in obese and insulin resistant mice identify TMAO and FMO3 as markers of altered metabolism [217]. Therefore, TMAO is generally associated with worsened insulin resistance and elevated blood glucose levels.

However, when TMAO effects on insulin signaling are investigated more closely in hepatocytes, adipocytes, and skeletal muscle, the molecular evidence is divided. While positive and negative TMAO effects on insulin signaling are reported in these tissues, the molecular mechanisms are best described in hepatic studies. Interestingly, TMAO downregulates the hepatic insulin signaling cascade [161,162], but beneficially reduces oxidative and ER stress associated with insulin resistance [117,136,165,171,195]. Other studies report no relationship between TMAO and insulin. Reducing TMAO levels by FMO3 and FMO5 knockdown in mice did not affect insulin resistance, nor did hepatic FMO3 knockdown alter AKT expression [135]. Diet-induced TMAO levels in healthy elderly subjects were not associated with blood glucose, insulin, HOMA-IR or hemoglobin A1c (HbA1c) measurements [143]. This result shows that TMAO may either not be causally related to poor blood glucose management or that TMAO effects differ in healthy versus diseased conditions. Therefore, while TMAO is generally associated with worsened insulin resistance and elevated blood glucose levels, there is molecular evidence for beneficial and deleterious TMAO effects on insulin signaling at target tissues.

Whole-body blood glucose management also hinges on pancreatic β-cell glucose tolerance and insulin secretion. As a nutrient sensing tissue, β-cells couple insulin secretion to elevated blood glucose levels. Impeded β-cell function, measured by in vivo glucose tolerance tests or the in vitro glucose stimulated insulin secretion assay, is a hallmark of diabetes. In high fat diet fed mice, TMAO supplementation increased serum levels to 17 μM and worsened glucose tolerance test results while TMAO reduction by FMO3 knockdown improved performance [48,161,242]. Although these studies highlight a connection between TMAO and blood glucose mismanagement, they do not directly measure β-cell function. Only one study on high fat diet-fed mice with a continuous TMAO intravenous pump suggests that TMAO ameliorated blood glucose levels via improved β-cell glucose tolerance and function [239]. Compared to vehicle controls, TMAO treated mice had better blood glucose and insulin levels during a glucose tolerance test [239]. Interestingly, TMAO showed no effect on glucose tolerance in healthy conditions, again supporting these pathogenic conditions elucidated a unique TMAO effect not mirrored in healthy contexts [239,242]. While this study approximated insulin secretion function in T2D mice, future β-cell culture experiments are needed to measure direct TMAO effects. Therefore, while TMAO has variable effects on blood glucose management such that it is generally linked to insulin resistance at the target tissue while it may benefit β-cell function under T2D conditions.

## 5. Conclusions

Although TMAO research in the human health context is young, we know that changes across the diet–microbiota–liver–kidney axis leads to serum accumulation, and that TMAO metabolic effects are context dependent. In CVD research, studies generally assert that TMAO is a deleterious dietary gut microbiome metabolite biomarker, although its molecular effects on vascular and cardiac tissue are mixed. TMAO’s role in tissues relating to metabolic diseases are further divided and may differ from its role in CVD [57]. While some data demonstrate that it is deleterious, others support beneficial TMAO effects. TMAO effects also differ between healthy and diseased conditions, such that metabolic stress seems to be a prerequisite for observable TMAO functions. Using such pathologic models, TMAO effects are best defined for hepatocytes while future research is needed to clarify mechanisms involving pancreatic β-cells. Negative TMAO effects are mainly observed in patients and animal models of NAFLD, gallstone formation, CKD, cognitive diseases, and T2D by aggravating insulin resistance and impairing cellular functions. The positive TMAO effects generally involve anti-oxidative or anti-inflammatory effects observed at the tissue level, especially in hepatocytes, adipocytes, muscle tissue, and pancreatic β-cells under stress from over-nutrition models. When considering its effects on blood glucose management, it is unclear if it improves or worsens insulin resistance overall. This debate between positive and negative TMAO effects calls for more research to better connect direct cellular mechanisms to clinical outcomes. It must be noted that the studies discussed here investigated TMAO effects via various research models. Studies induced elevated TMAO levels by altering the dietary substrate load, the microbiome TMA production, or hepatic FMO3 TMAO production. Other studies measure elevated TMAO levels in animal models using dietary or genetically mutant animals originally established to model metabolic diseases. Therefore, these models could alter metabolic tissues in other more direct ways that may overshadow or compound TMAO effects. Since interpreting causality in these studies in fraught with concerns, future TMAO research should of course consider clinical relevance of the research design closely. Since TMAO is an indicator of excess dietary consumption of choline and related compounds, future studies should continue to explore TMAO effects within the context of over-nutrition associated metabolic diseases contributing to our global health challenge.
